# Predicting field-goal success according to offensive, defensive and contextual variables in elite men’s wheelchair basketball

**DOI:** 10.1371/journal.pone.0244257

**Published:** 2021-01-07

**Authors:** John W. Francis, Alun J. Owen, Derek M. Peters

**Affiliations:** 1 School of Sport and Exercise Science, University of Worcester, Worcester, United Kingdom; 2 Faculty of Engineering, Environment and Computing, Coventry University, Coventry, United Kingdom; 3 School of Allied Health and Community, University of Worcester, Worcester, United Kingdom; Universidade de Tras-os-Montes e Alto Douro, PORTUGAL

## Abstract

The purposes of this study were to (i) develop a field-goal shooting performance analysis template and (ii) explore the impact of each identified variable upon the likely outcome of a field-goal attempt using binary logistic regression modelling in elite men’s wheelchair basketball. First, a field-goal shooting performance analysis template was developed that included 71 Action Variables (AV) grouped within 22 Categorical Predictor Variables (CPV) representing offensive, defensive and game context variables. Second, footage of all 5,105 field-goal attempts from 12 teams during the men’s 2016 Rio De Janeiro Paralympic Games wheelchair basketball competition were analysed using the template. Pearson’s chi-square analyses found that 18 of the CPV were significantly associated with field-goal attempt outcome (p < 0.05), with seven of them reaching moderate association (Cramer’s V: 0.1–0.3). Third, using 70% of the dataset (3,574 field-goal attempts), binary logistic regression analyses identified that five offensive variables (classification category of the player, the action leading up to the field-goal attempt, the time left on the clock, the location of the shot, and the movement of the player), two defensive variables (the pressure being exerted by the defence, and the number of defenders within a 1-meter radius) and 1 context variable (the finishing position of the team in the competition) affected the probability of a successful field-goal attempt. The quality of the developed model was determined acceptable (greater than 65%), producing an area under the curve value of 68.5% when the model was run against the remaining 30% of the dataset (1,531 field-goal attempts). The development of the model from such a large sample of objective data is unique. As such it offers robust empirical evidence to enable coaches, performance analysts and players to move beyond anecdote, in order to appreciate the potential effect of various and varying offensive, defensive and contextual variables on field-goal success.

## Introduction

Previous shooting research in wheelchair basketball has focused mainly on free-throw shooting [1–4], due we would argue, to the greater consistency and accuracy of variables to be measured in the ‘controlled’ setting enabling the use of statistical analyses requiring repeated measures assumptions to be met, and with fewer extraneous factors to consider, rather than because of its importance within a game. Indeed, field-goal attempts equate to the largest number of shot attempts during elite wheelchair basketball games with an average of between 57 and 64 per game from data obtained from the 2008 Paralympics in Beijing (China), the 2010 World Wheelchair Basketball Championships in Birmingham (UK), the 2015 European Wheelchair Basketball Championships in Worcester (UK) and the 2016 Paralympics in Rio de Janeiro (Brazil) compared to an average of between only 11 and 16 free throw attempts per game [5–8].

Field-goal shooting has been highlighted as one of the fundamental technical skills required by elite wheelchair basketball players [9,10] and Francis et al. [11] recently emphasised the importance of the offensive player taking less pressurised shooting opportunities to increase their shooting efficiencies. Although, such studies provide an initial overview of tactical considerations when field-goal shooting in wheelchair basketball, more specific knowledge is required to identify the key determinants of field-goal shooting success to advance coaching and game training practise.

In contrast to wheelchair basketball, within the last eight years, there have been several studies published attempting to identify the key components of effective field-goal shooting in running basketball. Skinner [12], for example, developed four predictive models to examine the effects of the shooter’s sequence, shot clock time remaining, shooter’s sequence from a turnover, and the shooting rates of optimal shooters on the success of a shot attempt in the NBA. However, each model contained only one single predictor and the parameter estimates from these singular predictive models were then collated to provide an overall expected point per possession score. Furthermore, the influence of the defence on the quality of the shot outcome was not considered.

Gorman and Maloney [13], in contrast, examined the change in a shooter’s execution when a defender was added to a shot attempt. Through analysing four field-goal shot types, the study found the presence of a defender resulted in a decreased shooting success of 20% as well as a faster shot action, a longer time spent in the air and a longer flight time of the ball. In both of these studies, however, the interactive effects of each action variable were not explored, therefore, the dynamic interactions that occur in basketball were not examined and the information does not explain how a player can achieve a higher probability of field-goal success when up against a variable defence.

More recently, Gómez et al. [14] used binary logistic regression modelling to highlight that field-goal success in running basketball is influenced by several action variables. By grouping offensive and defensive action variables into six categories and recording the sequential nature for each field-goal attempt, the study identified that during balanced games (difference between 0 to 9 points), shooting distance and shooting zone were found to be significant action variables, with closer field-goal attempts resulting in higher success. Whilst in unbalanced games (difference above 10 points), fewer passes and short possession durations were found to significantly reduce field-goal success. The research concluded therefore that field-goal success is dependent on offensive, defensive and contextual action variables, which were similar to those suggested as important components by both Skinner [12] and Gorman and Maloney [13].

These research findings could apply to wheelchair basketball field-goal shooting because the fundamental shooting principles of running basketball and wheelchair basketball are the same [15]. The improvements seen in NBA players’ field-goal shooting over past seasons as a result of increased focus by researchers and staff were identified in the works of Goldsberry [16], Chang et al. [17] and Shortridge et al. [18], and were illustrated at the end of season statistical reports [19,20]. The inclusion of offensive, defensive and contextual shooting variables, instead of using box-score frequency count data, could aid wheelchair basketball coaches, performance analysts and players future decision-making process around training as well as offensive and defensive strategies through capturing broader and more contextually relevant data. The purposes of this study, therefore, were to (i) develop a field-goal shooting performance analysis template and (ii) explore the impact of each identified variable upon the likely outcome of a field-goal attempt using binary logistic regression modelling in elite men’s wheelchair basketball.

## Materials and methods

### Sample

The sample consisted of all 5,105 field-goal attempts taken during the men’s wheelchair basketball competition at the 2016 Rio de Janeiro Paralympic Games in Brazil (Table 1). Following ethical approval from the University Ethics and Research Governance Committee, written voluntary informed consent was obtained from the Performance Director of one of the 12 competing nations at the 2016 Rio de Janeiro Paralympic Games granting permission to use the national team’s footage archive from the 2016 Paralympic Games.

**Table 1:** Field-goal attempts and shooting efficiencies in competition ranking order for each nation.

Ranking	Nation	Games Played	Games Won	Total Field-Goal Attempts	Successful Field-Goal Attempts	Unsuccessful Field-Goal Attempts	Overall Shooting Efficiency
1^st^ (Gold)	USA	8	8	512	265	247	51.8%
2^nd^ (Silver)	Spain	8	6	498	231	267	46.4%
3^rd^ (Bronze)	GB	8	6	525	256	269	48.8%
4^th^	Turkey	8	5	472	226	246	47.9%
5^th^	Brazil	7	3	427	186	241	43.6%
6^th^	Australia	7	4	416	190	226	45.7%
7^th^	Netherlands	7	3	431	161	270	37.4%
8^th^	Germany	7	2	439	181	254	41.2%
9^th^	Japan	6	2	365	145	220	39.7%
10^th^	Iran	6	2	348	156	192	44.8%
11^th^	Canada	6	2	345	114	231	33.0%
12^th^	Algeria	6	0	327	100	227	30.6%

### Variables, performance analysis template, validity & reliability

Due to the lack of a suitable performance analysis data collection tool, the nine-stage process completed by Francis et al. [21] for developing a valid and reliable sports performance analysis data collection tool was followed. First, a proposed list of 84 action variables within 23 categories, was developed from the previous literature and our experience in wheelchair basketball. Second, following ethical approval from the University Ethics & Research Governance Committee and after receipt of written informed consent, this proposed list was discussed with a focus group consisting of three elite wheelchair basketball coaches (Coach one: 20 years’ experience; Coach two: 19 years’ experience; Coach three: 19 years’ experience) and a member of support staff from an elite wheelchair basketball team (3 years’ experience). Third, following the focus group’s discussion, the list of action variables was revised, with the Number of Passes category being removed, the Defensive System category being replaced with the Number of Defenders category, and the number of action variables within the Defensive Pressure category reduced from six to five all to more concisely focus on the actions either being performed during or affecting the final field-goal attempt. Player classification was also identified as too discriminating in terms of its categorisation of the players that was not deemed reflective of how players play or the ways in which they are strategized to play within matches e.g. low point category players (1.0 and 1.5 classification) were coached, treated and expected to perform the same roles and actions as each other within games, as were mid-point category players (2.0 to 3.0 classification) and as were high point category players (3.5 to 4.5 classification). Players were therefore identified into Classification Category rather than identified by their individual classification. Fourth, operational definitions for the 71 remaining action variables in the 22 categories were developed and presented to the experts during a second focus group discussion. Fifth, amendments were made to operational definitions of the action variables in the Shot Positioning, Shot Movement and Defensive Pressure categories. Sixth, video clips of each agreed action variable were created to establish content validity. The experts agreed on the final list of 71 action variables and operational definitions in the 22 categories and assigned each to either an offensive, defensive, or contextual category. The title of each category is referred to as a Categorical Predictor Variable (CPV) and the variables within each CPV are referred to as Action Variables (AV). Further details of the 22 CPVs, including the action variables and operational definitions, are provided in SUP1 File.

**Fig 1:**
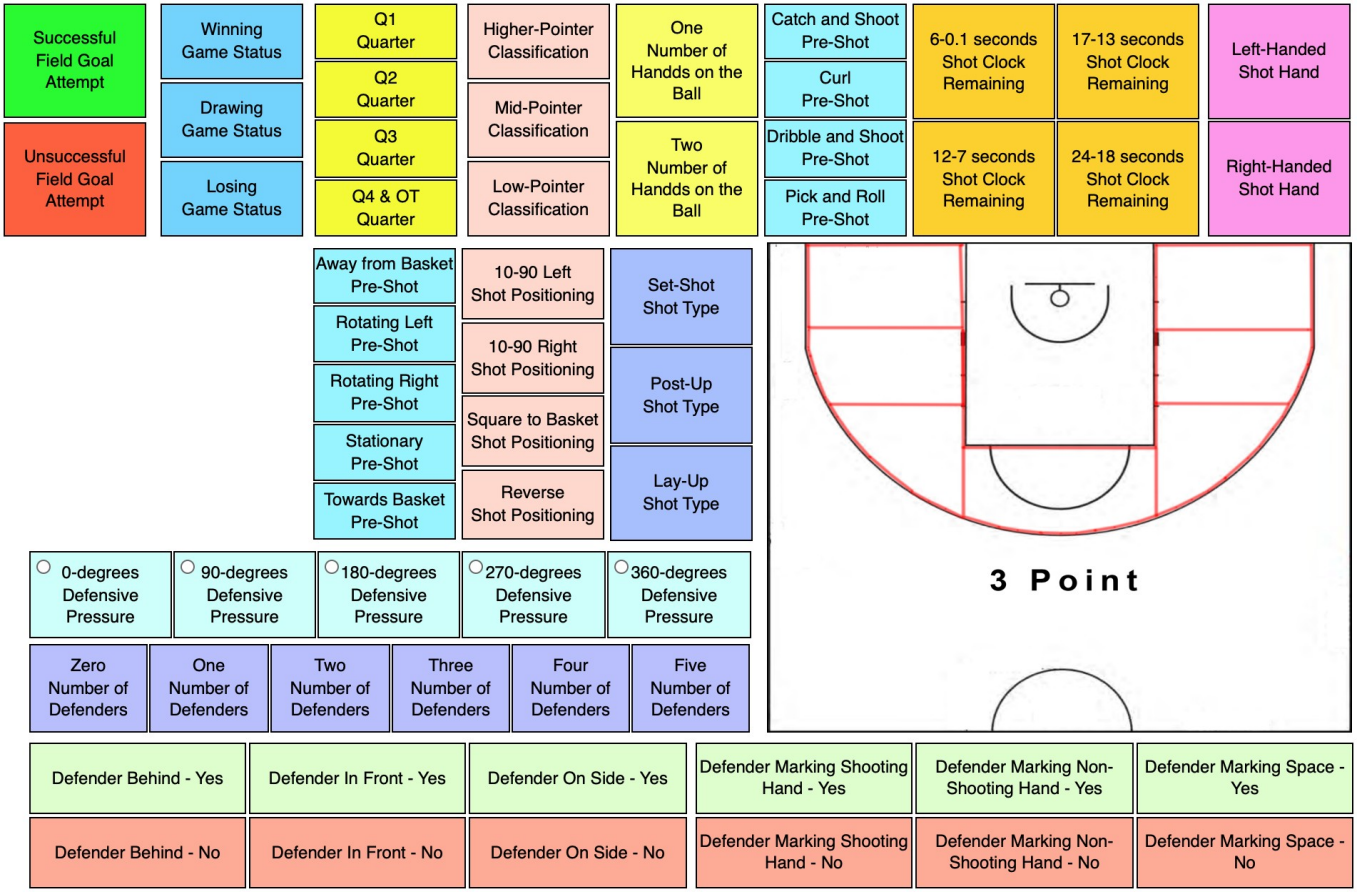
Field-goal attempt sports performance analysis template for wheelchair basketball.

Following the validation process (stages one to six above), a performance analysis template was created in SportsCode Elite Version 11 during stage seven. The template underwent two pilot tests on a randomly selected set of elite wheelchair basketball field-goal attempts taken from two pre-tournament games held in 2015. As a result of these pilot tests, the buttons were resized and positioned in their category group (Fig 1).

At stage eight, the developed field-goal attempt template was subjected to intra-observer reliability assessments. Percentage Error [22] values were used to assess all 22 CPVs, Cohen’s Kappa [23] was used to assess the nominal CPVs (17 CPVs) and a Weighted Cohen’s Kappa for the ordinal CPVs (five CPVs) [24]. A total of 200 field-goal shot attempts taken during the 2015 European Wheelchair Basketball Championships were observed on two separate occasions (Ob1 and Ob2), four weeks apart. The 22 categories reported percentage error values of below five per cent error and were either in perfect (k = 1.00) or almost perfect agreement (k = 0.91–0.97) between the first (Ob1) and second observation (Ob2) (Table 2). Where a discrepancy was identified, the specific video clip of the shot attempt was re-observed to create a final agreed observation (Ob3).

**Table 2:** Intra-observer and inter-observer reliability test results for the field-goal attempt template.

Category	CPV	Ob1 v Ob2		Ob3 v Ob4		Ob3 v Ob5		Ob3 v Ob4 v Ob5	
		%	k	%	k	%	k	%	k
Offensive	Classification Category	0.0	1.00	0.0	1.00	0.0	1.00	0.0	1.00
	Field-Goal Attempt Outcome	0.0	1.00	0.0	1.00	0.0	1.00	0.0	1.00
	Number of Hands on the Ball	0.0	1.00	0.0	1.00	0.0	1.00	0.0	1.00
	Shot Point	0.0	1.00	0.0	1.00	0.0	1.00	0.0	1.00
	Pre Shot	1.9	0.97	1.9	0.97	0.0	1.00	3.8	0.96
	Shot Clock Remaining	0.0	1.00	0.0	1.00	0.0	1.00	0.0	1.00
	Shot Hand	0.0	1.00	0.0	1.00	0.0	1.00	0.0	1.00
	Shot Location	0.0	1.00	0.0	1.00	0.0	1.00	0.0	1.00
	Shot Movement	3.8	0.94	1.9	0.97	1.9	0.97	1.9	0.98
	Shot Positioning	3.8	0.92	1.9	0.96	0.0	1.00	1.9	0.97
	Shot Type	0.0	1.00	0.0	1.00	0.0	1.00	0.0	1.00
Defensive	Defender Behind	1.9	0.95	3.8	0.89	3.8	0.89	3.8	0.93
	Defender In Front	3.8	0.92	1.9	0.96	1.9	0.96	1.9	0.97
	Defender Marking Non-Shooting Hand	1.9	9.44	1.9	0.95	1.9	0.95	3.8	0.93
	Defender Marking Shooting Hand	1.9	0.96	1.9	0.96	0.0	1.00	1.9	0.97
	Defender Marking Space	3.8	0.90	0.0	1.00	0.0	1.00	0.0	1.00
	Defender On Side	3.8	0.91	0.0	1.00	3.8	0.90	3.8	0.94
	Defensive Pressure	3.8	0.94	1.9	0.97	3.8	0.94	3.8	0.96
	Number of Defenders	0.0	1.00	0.0	1.00	0.0	1.00	0.0	1.00
Contextual	Game Status	0.0	1.00	0.0	1.00	0.0	1.00	0.0	1.00
	Quarter	0.0	1.00	0.0	1.00	0.0	1.00	0.0	1.00

Note: Ob1: First author’s first observation; Ob2: First author’s second observation; Ob3: First author’s agreed observation; Ob4: Coach’s observation; Ob5: Performance analyst intern’s observation.

Ninth, one wheelchair basketball coach (Ob4) and a performance analyst intern (Ob5) completed an inter-observer reliability test. Before completing the inter-observer tests, the coach and intern familiarised themselves with the developed template. The individuals used a trial shooting sample from a men’s warm-up game that consisted of 100 field-goal attempts. The inter-observer reliability tests were not completed until both observers felt they were able to accurately record the shot attempts. Comparing Ob3 against Ob4 and Ob3 against Ob5 reported acceptable percentage error values (less than five per cent) and perfect or almost perfect agreement (k> 0.80) for the 22 categories. As a final assessment, Percentage Error values and a Fleiss’ Kappa [25] was used to compare all three observations. All CPV’s reported less than five per cent error and perfect or almost perfect agreement coefficients (Table 2). The intra-observer and inter-observer reliability results highlight the observers were able to accurately record the specific action variables that occurred during each field-goal attempt when using the designed template.

### Data collection and handling procedure

The obtained video footage was filmed from the half-way line in an elevated position and provided two half-court perspectives with an overlay of the time clock and current scoreboard. The 5,105 field-goal attempts were analysed over three months by the lead author using the template developed above [26].

Following the completion of the data collection in SportsCode (Agile Sports Technologies, Inc.), the field-goal attempt data were exported into a Microsoft Excel spreadsheet using the ‘Sorter’ function in SportsCode. The data set consisted of 5,105 rows of data, with each row representing the sequence of actions leading up to a single field-goal attempt. The field-goal shooting dataset consisted of 24 columns, which included the 21 exploratory CPVs, the dependent CPV (Field-Goal Attempt Outcome) and a column entitled Shot Number and Game ID. The dataset was subjected to data checking procedures to identify any discrepancies within the data. Within the Number of Defenders CPV, no occurrences of ‘Four’ or ‘Five’ defenders were found; these two action variables were removed from further analysis. The Excel file was converted into a CSV file and subjected to statistical analysis procedures similar to those used previously (please refer to Francis et al. [11]).

### Statistical methods

#### Stage 1: Exploring the association between individual Categorical Predictor Variables (CPV) and field-goal attempt outcome

Pearson’s Chi-square analysis was carried out to determine if there was a statistically significant association between each CPV and field-goal attempt outcome. Cramer’s V (φ_c_) was used to determine the degree of any observed associations and is expressed as a weak association (φ_c_ < 0.1), moderate association (φ_c_ between 0.1 and 0.3) or strong association (φ_c_ between 0.3 and 0.5) [23].

#### Stage 2: Modelling the effect of CPV’s on field-goal attempt outcome

Following an assessment of inter-association and multicollinearity, a binary logistic regression model was developed with the field-goal attempt outcome as the binary (successful/unsuccessful) dependent variable. This model has been widely used in the sports performance analysis research literature, and whilst this assumes independence between successive field-goals attempts within a match, we consider this to be reasonable due to the wide varying nature of each attempt described by 22 CPVs (covering 71 different action variables). An automated stepwise approach was undertaken when selecting suitable CPVs as explanatory variables in the model. The model was developed using 70% of the dataset (3,574 shots) [27]. The use of this binary regression model allowed changes in individual contributions of each action variable within a CPV to be compared to an identified baseline action variable within the CPV. The baseline category within each CPV against which all other action variables were compared was identified as the most logical highest likelihood of success e.g. ‘2 point-centre-near’, ‘1^st^ ranked team’, ‘zero defenders’, ‘high-pointer’, ‘stationary’, ‘catch & shoot’ and ‘0-degrees’ of defensive pressure. We included ‘0.1–6 seconds’ in the Shot Clock Remaining as the Baseline Category as the gives the offensive team the maximum time to set up their best shooting opportunity.

These differences were calculated via the estimated regression coefficients and their standard error values along with their p-values, Odds Ratio (OR) values and their 95% confidence intervals (CIs). The estimated regression coefficients demonstrated the action variables’ contribution to the prediction of the outcome (field-goal attempt success), with a positive estimated regression coefficient being associated with an increase in the odds of a successful field-goal attempt compared to the baseline category. The OR represents a measure of association between the explanatory CPVs and field-goal attempt outcome. If an OR for an action variable is greater than one, this means that if this action variable occurs in a CPV it is associated with higher odds of field-goal success. Whereas, if an OR of less than one is found for an action variable, this describes a negative relationship, and means that if this action variable in a CPV occurs it is associated with lower odds of field-goal success.

The fit of the developed model was determined by a Hosmer and Lemeshow’s [28] Goodness of Fit test and a Log-Likelihood Ratio Test (LRT) [29]. Whilst, the model’s ability to accurately predict out of sample field-goal outcome was determined by calculating the area under the receiver operating characteristic (ROC) curve [30], using the remaining 30% of data (1,531 shots). The area under the ROC curve measures the sensitivity and specificity of the developed model, with a potential range of zero to one with the value representing the discriminant power of the model i.e. if the model is able to predict the dependent variable outcome with 50% accuracy, the value would be 0.5, with 75% accuracy it would be 0.75 etc. [30].

Statistical analyses, for both stages, were undertaken using the R statistical software [31], version 3.6.3, along with the following R packages: “car” [Companion to Applied Regression [32]], “caret” [Classification and Regression Training [33]], “scales” [Scale Functions for Visualization [34]] and “ROCR” [35]. All statistical tests were conducted at the 5% level of significance.

## Results

### Stage 1: Exploring the association between individual Categorical Predictor Variables (CPV) and field-goal attempt outcome

Table 3 summarises the results of Pearson’s Chi-square tests of association between the 21 CPVs and Field-Goal Attempt Outcome. Significant associations were observed for 18 CPVs. The Shot Location CPV reported the lowest p-value and largest degree of association of any observed association (χ2(9) = 231.02, p < 0.001, φ_c_ = 0.213, medium). Whilst the Defender On Side CPV reported the highest p-value and smallest degree of association (χ2(1) = 0.03, p = .872, φ_c_ = 0.003, weak). Of the 18 significant associations, seven demonstrated a moderate association (φ_c_ between 0.1 and 0.3) and 11 demonstrated a weak association (φ_c_ < 0.1).

**Table 3:** Pearson Chi-square tests of association between each CPV and field-goal attempt outcome.

Category	CPV	*χ*^2^	df	*P*	Cramer’s V	
					(φ_c_)	
Offensive	Shot Location	231.02	9	<0.001***	0.213	Medium
	Shot Type	150.68	2	<0.001***	0.172	Medium
	Shot Point	108.93	1	<0.001***	0.147	Medium
	Shot Clock Remaining	103.86	3	<0.001***	0.143	Medium
	Pre Shot	51.15	3	<0.001***	0.1	Medium
	Shot Movement	50.33	4	<0.001***	0.099	Weak
	Shot Positioning	46.59	3	<0.001***	0.096	Weak
	Classification Category	12.48	2	0.002**	0.049	Weak
	Shot Hand	6.6	1	0.010**	0.037	Weak
	Number of Hands	6.23	1	0.013*	0.036	Weak
Defensive	Number of Defenders	68.09	3	<0.001***	0.115	Medium
	Defender In Front	40.43	1	<0.001***	0.089	Weak
	Defensive Pressure	30.9	4	<0.001***	0.078	Weak
	Defender Marking Shooting Hand	19.31	1	<0.001***	0.062	Weak
	Defender Behind	5.61	1	0.018*	0.034	Weak
	Defender Marking Space	4.52	1	0.034*	0.03	Weak
	Defender Marking Non-Shooting Hand	0.54	1	0.461	0.011	Weak
	Defender On Side	0.03	1	0.872	0.003	Weak
Contextual	Ranking	73.76	11	<0.001***	0.12	Medium
	Game Status	34.72	2	<0.001***	0.082	Weak
	Quarter	1.87	3	0.599	0.019	Weak

Note:

*p < 0.05,

**p < 0.01,

***p < 0.001.

### Stage 2: Modelling the effect of CPV’s on field-goal attempt outcome

Multicollinearity was detected between Shot Point and Shot Location (Variance Inflation Factor: 1376.44) Two binary logistic regression models were built (Model 1: Field-Goal Attempt Outcome and Shot Point; Model 2: Field-Goal Attempt Outcome and Shot Location), and the Shot Location model provided better predictive performance (Akaike Information Criterion Values: Model 1: 7237.5; Model 2: 7104.7) [36]. The remaining 17 statistically significant variables (omitting Shot Point) were inputted into an automated stepwise binary logistic model building process. The final model consisted of the following eight CPVs: Shot Location, Shot Clock Remaining, Ranking, Number of Defenders, Classification Category, Shot Movement, Pre Shot and Defensive Pressure. The predictive accuracy of the model for shooting success found no evidence of poor fit when the Hosmer-Lemeshow Goodness of Fit Test was used (χ2(8) = 9.967, p = .267).

Table 4 summarises the results of the final model, with Chi-squared likelihood ratio tests showing that these CPVs all uniquely contributed to the model: Shot Location (χ2 (9) = 75.38, p < .001), Shot Clock Remaining (χ2 (3) = 31.64, p < 001), Ranking (χ2 (11) = 47.33, p < .001), Number of Defenders (χ2 (3) = 19.45, p < .001), Classification Category (χ2 (2) = 35.63, p < .001), Shot Movement (χ2 (4) = 17.85, p < .001), Pre Shot (χ2 (2) = 12.81, p = .005) and Defensive Pressure (χ2 (3) = 8.30, p = .081), that whilst not presenting a statistically significant contribution, improved the model fit when it was retained. The regression equation derived from the model was then used for predicting the accuracy of the binary logistic regression model against the 30% out of sample testing data (1,531 shots). An area under the ROC curve value of 0.685 was established for the model when predicting field-goal attempt outcome within the out of sample testing data, identifying that the model was able to accurately classify field-goal outcome 68.5% of the time, which represents a good level of discriminant capacity(>0.65%) [28,30].

**Table 4:** Final model illustrating the frequency counts (n) and percentage success (Suc) from the 70% sample (3,574 field-goal attempts), likelihood ratio test (LRT) values, estimated regression coefficients, standard errors, p-values and ORs for the intercept variable and each action variable within a CPV.

Category	CPV	Action Variable	n	Suc	LRT	Estimate	SE	z	*p*	OR (OR 95% CI)
Intercept						0.701	0.186	3.769	.001***	2.016 (1.400–2.905)
Offensive	Shot Location	2 Point—Centre—Near ^BC^	1441	54.55%	χ2 (9) = 75.38; *p <* 0.001***	0	N/A			
		2 Point—Centre—Mid	351	39.03%		-0.375	0.13	-2.891	.004**	0.688 (0.533–0.885)
		2 Point—Centre—Long	328	32.92%		-0.644	0.139	-4.617	< .001***	0.525 (0.399–0.689)
		2 Point—Left—Base	146	41.10%		-0.083	0.188	-0.44	0.66	0.920 (0.635–1.329)
		2 Point—Left– 45	254	38.98%		-0.304	0.151	-2.017	.044*	0.738 (0.548–0.990)
		2 Point—Left—Elbow	177	32.30%		-0.615	0.181	-3.394	.001***	0.540 (0.377–0.768)
		2 Point—Right—Base	91	43.96%		0.034	0.231	0.146	0.884	1.034 (0.655–1.624)
		2 Point—Right– 45	261	45.21%		-0.054	0.148	-0.366	0.714	0.947 (0.709–1.265)
		2 Point—Right—Elbow	212	36.79%		-0.423	0.164	-2.571	.010*	0.655 (0.473–0.903)
		3 Point	313	24.60%		-1.113	0.157	-7.068	< .001***	0.329 (0.240–0.446)
	Classification Category	High-Pointer ^BC^	1932	45.65%	χ2 (2) = 35.63; *p <* 0.001***	0	N/A			
		Mid-Pointer	1303	43.21%		-0.241	0.087	-2.756	< .001***	0.786 (0.661–0.932)
		Low-Pointer	339	33.92%		-0.8	0.139	-5.763	.006**	0.449 (0.341–0.588)
	Shot Clock Remaining	0.1–6 seconds ^BC^	1001	34.47%	χ2 (3) = 31.64; *p <* 0.001***	0	N/A			
		12–7 seconds	1376	45.51%		0.246	0.09	2.722	.006**	1.279 (1.072–1.527)
		17–13 seconds	910	50.33%		0.499	0.102	4.874	< .001***	1.647 (1.348–2.014)
		24–18 seconds	287	59.93%		0.693	0.156	4.456	< .001***	2.000 (1.476–2.716)
	Shot Movement	Stationary ^BC^	952	45.80%	χ2 (4) = 17.85; *p* = 0.001**	0	N/A			
		Away From Basket	348	39.08%		-0.293	0.134	-2.183	.029*	0.746 (0.573–0.970)
		Rotating Left	768	25.29%		-0.137	0.113	-1.21	0.226	0.872 (0.699–1.088)
		Rotating Right	350	38.86%		-0.159	0.138	-1.154	0.249	0.853 (0.651–1.116)
		Towards Basket	1156	50.26%		0.184	0.107	1.717	0.086	1.202 (0.974–1.483)
	Pre Shot	Catch & Shoot ^BC^	2193	46.01%	χ2 (3) = 12.81; *p* = 0.005**	0	N/A			
		Curl	42	61.90%		0.098	0.334	0.295	0.768	1.103 (0.579–2.162)
		Dribble & Shoot	1222	37.89%		-0.271	0.084	-3.221	.001**	0.763 (0.646–0.899)
		Pick n Roll	117	52.99%		0.245	0.208	1.182	0.237	1.278 (0.852–1.924)
Defensive	Number of Defenders	Zero ^BC^	700	55.14%	χ2 (3) = 19.45; *p <* 0.001***	0	N/A			
		One	2183	39.62%		-0.508	0.121	-4.207	< .001***	0.601 (0.474–0.762)
		Two	611	44.19%		-0.342	0.164	-2.08	0.038*	0.711 (0.515–0.980)
		Three	80	48.75%		-0.065	0.317	-0.204	0.839	0.937 (0.504–1.756)
	Defensive Pressure	0-degrees ^BC^	1308	47.94%	χ2 (4) = 8.30; *p* = 0.081	0	N/A			
		90 degrees	1493	41.39%		0.036	0.105	0.347	0.729	1.037 (0.845–1.274)
		180 degrees	497	40.64%		-0.165	0.141	-1.172	0.241	0.848 (0.643–1.117)
		270 degrees	234	41.03%		-0.418	0.197	-2.123	.034*	0.658 (0.447–0.967)
		360 degrees	42	40.48%		-0.724	0.405	-1.788	0.074	0.485 (0.216–1.064)
Contextual	Ranking	1st ^BC^	367	51.77%	χ2 (11) = 47.33; *p <* 0.001***	0	N/A			
		2^nd^	331	50.45%		-0.2	0.164	-1.223	0.221	0.818 (0.593–1.128)
		3^rd^	354	46.89%		-0.09	0.157	-0.575	0.565	0.913 (0.671–1.243)
		4^th^	332	50.90%		-0.065	0.164	-0.394	0.694	0.937 (0.679–1.294)
		5^th^	291	40.55%		-0.421	0.169	-2.495	.013*	0.657 (0.471–0.913)
		6^th^	294	48.64%		-0.042	0.168	-0.253	0.801	0.959 (0.690–1.332)
		7^th^	313	36.74%		-0.54	0.165	-3.277	.001**	0.583 (0.421–0.804)
		8^th^	310	40.97%		-0.362	0.166	-2.181	.029*	0.696 (0.502–0.963)
		9^th^	260	37.69%		-0.509	0.174	-2.92	.004**	0.601 (0.427–0.845)
		10^th^	255	44.71%		-0.322	0.18	-1.796	0.072	0.724 (0.509–1.029)
		11^th^	238	33.19%		-0.803	0.184	-4.36	< .001***	0.448 (0.311–0.641)
		12^th^	229	32.31%		-0.692	0.189	-3.651	< .001***	0.501 (0.344–0.724)

Note:

*p < 0.05,

**p < 0.01,

***p < 0.001;

B, estimate coefficient; SE, standard error; OR, odds ratios; CI, confidence intervals; BC = baseline categories.

Table 4 also shows the individual parameter estimates for each action variable, within a CPV, and their associated standard errors (SE), Z-statistics, p-values Odds Ratios (OR) and 95% CIs for the Odds Ratios. Using the OR per CPV derived from the model, the highest field-goal success occurred when an attempt was made from near or the right base location (OR:1.034) in the first six seconds of a 24-second possession (OR:2.000) by a high point player (OR:1.273) from the USA, marked by no defenders, moving towards the basket (OR:1.202) from a ‘Pick n Roll’ (OR:1.278) when he experienced a defensive pressure of 90 degrees (OR:1.037) at the time of the attempt. Whilst the lowest shooting success occurred when a three-point attempt was made (OR: 0.329) in the last six seconds of the 24-second possession by a low-point player (OR: 0.449) from the 11^th^ ranked team (OR: 0.448) moving away from the basket (OR: 0.746) after they had finished dribbling the ball (OR: 0.763) whilst being marked by one defender (OR: 0.601) and experiencing pressure across his entire cylinder (OR: 0.485).

Further insights from the data can also be obtained by comparing one action variable with another action variable within the same CPV to examine their effects on the likelihood of field-goal success. These are presented below for the significant offensive, defensive and context categories:

#### Offensive category

**Fig 2:**
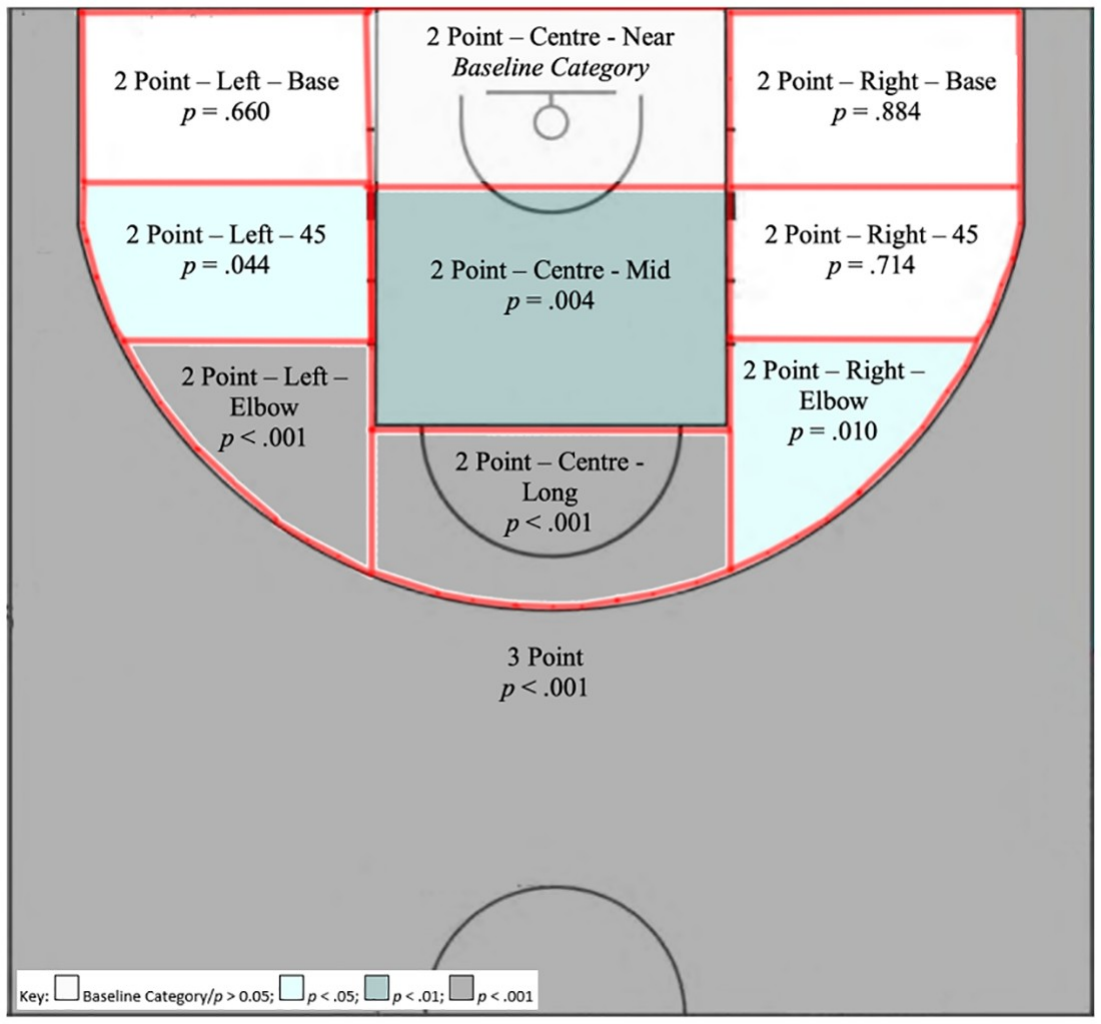
Field-goal attempt locations indicating significant differences from the baseline category of ‘2 Point—Centre—Near’.

Shot Location: When included in the model, Shot Location reported the largest degree of association with Field-Goal attempt success. The data in Table 4 showed that as distance away from the basket increased the likelihood of achieving field-goal success decreased. In particular, when a field-goal attempt was made from the ‘2 Point—Centre—Mid’ (OR: 0.688; 95% CI: 0.533–0.885; p = .004) location the odds of success were 31% lower than when an attempt was made from the baseline category of ‘2 Point—Centre—Near’. The odds of success were 11% lower still when the distance increased from the ‘2 Point—Centre—Mid’ location to the ‘2 Point—Centre—Long’ (OR: 0.525; 95% CI: 0.399–0.689; p < .001) location. Whilst an attempt from the 3 Point’ (OR: 0.329; 95% CI: 0.240–446; p < .001) location in comparison to an attempt from the ‘2 Point—Centre—Near’ location resulted in a decrease in the odds of shooting success by 67%, the effect of the angle from which the three-point field-goal was attempted cannot be determined as all field-goals beyond the three-point line were categorised only as being from the ‘3 Point’ location (Fig 2).

Compared to the baseline category, field-goal success was also significantly lower when the attempt was taken from an angle e.g. ‘2 Point—Left—Elbow’ (OR: 0.540; 95% CI: 0.377–0.768; p = .001, 46% lower), ‘2 Point—Right—Elbow’ (OR: 0.655; 95% CI: 0.473–0.903; p = .010, 34% lower) and ‘2 Point—Left— 45’ (OR: 0.738; 95% CI: 0.548–0.990; p = .044, 26% lower), with the differences most pronounced at each ‘Elbow’ position and with more areas identified as lowering field-goal success on the left side of the court (Fig 2).

Classification Category: Table 4 illustrates that when a ‘Mid-Pointer’ attempted a field-goal their odds of success were significantly lower (by 21%) than when a ‘High-Pointer’ attempted an identical shot (OR: 0.786; 95% CI: 0.662–0.932; p < .001). Whilst, if a ‘Low-Pointer’ attempted a field-goal, their odds of success were 43% lower in comparison to an identical attempt by a ‘High-Pointer’ (OR: 0.572; 95% CI: 0.433–0.751; p < .001). The distribution of field-goal attempts made also clearly identifies that an attempt by a ‘Low-Pointer’ occurs far less often than the other two classification categories. The frequency count of field-goal attempts and shooting efficiency increased as the classification category changed from ‘Low-Pointer’ to ‘Mid-Pointer’ to ‘High-Pointer’.

Shot Clock Remaining: When included in the model, Shot Clock Remaining was significantly associated with field-goal success. The data highlighted the odds of success were doubled when a field-goal was attempted with ‘24–18 seconds’ remaining (OR: 2.000; 95% CI: 1.476–2.716; p < .001) instead of the last ‘0.1–6 seconds’ on the clock. Indeed, as each six-second period elapses, whilst the odds of field-goal success continually decrease (17–13 seconds: OR: 1.647; 95% CI: 1.348–2.014; p < .001; 12–7 seconds: OR: 1.279; 95% CI: 1.072–1.527; p = .004) the odds of success in each time period were significantly higher than field-goals attempted within the final six seconds.

Shot Movement: With regards to the shooting player’s movement, the odds of field-goal success were significantly lower (25%) when an attempt was made while moving ‘Away From Basket’ (OR: 0.746; 95% CI: 0.573–0.970; p = .029) in comparison to the baseline category of ‘Stationary’. ‘Towards Basket’ whilst not quite reaching statistical significance (p = .086), offered a greater percentage success rate (50.26%) and higher odds of success (OR 1.202) than shooting from ‘Stationary’.

Pre Shot: The odds of field-goal success were only significantly reduced if the shooter’s actions were from a ‘Dribble & Shoot’ compared to the baseline category of ‘Catch & Shoot’ (OR: 0.763; 95% CI: 0.646–0.899; p < .001). This pre-shot action resulted in a 24% lower odds of field-goal success. Despite both ‘Curl’ and ‘Pick & Roll’ having substantially greater success percentages than the baseline category of ‘Catch & Shoot’ (61.9% and 52.99%), and OR above 1.0 (1.103 and 1.278) the frequencies of their occurrence (only 42 and 117) would have impacted upon the likelihood of these differences reaching statistical significance within the modelling process.

#### Defensive category

Number of Defenders: The odds of a field-goal attempt being successful were 40% lower when there was ‘One’ defender within one metre of the shooting player making either physical chair contact or visually engaging the shooter, compared to when there were no defenders within this distance (OR: 0.601; 95% CI: 0.474–0.762; p < .001). Whilst still resulting in significantly lower field-goal success compared to ‘Zero’ defenders, when ‘Two’ defenders (OR: 0.711; 95% CI: 0.515–0.980; p < .038) were within one meter, this appeared to lessen the negative impact on the field-goal success seen when only ‘One’ defender was present (i.e. 29% lower success compared to 40% lower success).

Defensive Pressure: The data highlighted the odds of success were significantly reduced when a shooting player faced defensive pressure of ‘Three’(OR: 0.658; 95% CI: 0.447–0.967; p = -.034) in comparison to facing ‘Zero’ defensive pressure. This meant that when 270 degrees of a shooting players sphere were disrupted by a defender compared to having zero degrees of their sphere disrupted, the odds of shooting success were 34% lower.

#### Contextual category

Ranking: Table 4 indicated that given a field-goal attempt in identical circumstances for all teams, there is no evidence to separate the teams ranked second, third or fourth from the top-ranked team (e.g. for team Ranked second, OR: 0.818; 95% CI: 0.593–1.128; p = 0.221). Whilst the data indicated lower-ranked teams predominantly (except 6^th^ placed Australia and 10^th^ placed Iran) are not as good at converting the same opportunities. The teams ranked 11th and 12th odds of field-goal success were significantly lower than those of the first-placed team by 45% and 49% respectively with the odds of success effectively halved (OR: 0.448 and 0.501).

## Discussion

The purposes of this study were to (i) develop a field-goal shooting performance analysis template and (ii) explore the impact of each identified variable upon the likely outcome of a field-goal attempt using binary logistic regression modelling in elite men’s wheelchair basketball. We have successfully developed and present a valid performance analysis template that we have also shown to be reliable to use. We recommend that this template form the basis for future field-goal performance analysis in wheelchair basketball in both applied and research contexts and at all levels, age groups and sexes. The template is also adaptable for use in the emerging different formats of the game e.g. 3-on-3. When the 17 individual significantly associated CPV (omitting Shot Point) were entered into a binary logistic regression model with field-goal success as the dependent variable, the final best-fitting model included only eight of them. These were the same five offensive, one defensive and one contextual CPVs that had previously identified medium association individually, plus an additional defensive CPV that, whilst not presenting a unique statistically significant contribution, improved the model fit, that being the spherical coverage of defensive pressure under which the shooting player was placed.

### Offensive implications

It is interesting to note that our findings indicated there were no significant reductions in field-goal success when attempts were made from the ‘Base’ (Left or Right) and also the ‘Right-45’ locations compared to an attempt from the ‘Near-Centre’ location. Within running basketball, field-goal attempts closer to the basket resulted in a higher success rate, however, the efficiency of attempts from further away from the basket were evenly distributed [18]. The differences observed here may be attributed to the surface area of a player in running basketball occupying a 1-square foot area whilst in wheelchair basketball a player’s chair typically occupies a 3-square foot area, subsequently affecting their ability to position themselves close to the basket if being defended by the opposition. Therefore, wheelchair basketball teams should look to prioritise these four locations as areas from which to make a field-goal shot attempt.

Our findings identified that field-goal attempts taken earlier in the possession resulted in significantly higher chances of success. This finding draws parallels to running basketball [12]. In the early stages of possession, defensive systems are unstable, favouring the offensive team to capitalise on their chances. This is due to the offence dictating play and the defence having less time to organise themselves to counter the threats posed. However, as more time elapses the balance favours the defensive team with offensive players having to take more rushed actions [37]. Similarly, previous research has demonstrated that taking a shot early in a possession is often made possible by disorganised and unbalanced defensive systems [38]. When a defensive team are unbalanced, the number of miss-matches, whereby high point players are being defended by lower point players increases due to a switching defence [39]. Therefore, facing a disorganised defensive team or miss-match situations favours the progression of the offensive team, allowing the team not only to dictate time but also space [40], resulting in players attempting their preferred shots early in a possession and potentially against less well-matched defenders. Teams should therefore focus on converting transitional offences into optimal shooting opportunities within the first six seconds of a possession. However, if a shot attempt early in a possession does not materialise, the model identifies that the focus should then shift to enabling a shot attempt during the 17 to 7 second period from one of the key identified shot locations. A possible way to achieve this is through maximising the two-man game in the base or 45 locations and creating miss-matches between defender and shooter.

Whilst unsurprisingly, the data show that the first ranked team had the superior field-goal shooting efficiency, it also reveals there were no differences in the shooting efficiencies of the top four ranked teams. There was evidence though that the bottom six teams have a significantly lower ability to convert similar shooting opportunities, except the 10^th^ placed team. Similar findings were found by Gryko et al. [41] in running basketball with the top eight teams being significantly different than the bottom eight teams in converting field-goal opportunities. Thus, despite the rank of the defensive team, the offensive team should look to devise strategies which maximise early field-goal attempts from the four key areas. The anomaly that is Iran seems reasonable, however, as high-point players from this team took 81% of the total team’s field-goal attempts across the tournament, which was the highest percentage for all teams, and congruent with the finding that both ‘Low-Pointer’ and ‘Mid-Pointer’ players had significantly lower chances of field-goal attempt success in comparison to ‘High-Pointer’ players.

The classification category pattern aligns with previous wheelchair basketball studies that highlighted high-point (3.5–4.5) players typically achieved higher field-goal shooting efficiencies [6]] Possible reasons for this could be due to the ability of a high-point player to engage the core and provide a more stable base of support and higher release point and angle when propelling the ball towards the basket. Whereas, if low-point and mid-point classified players are attempting to generate the necessary force to propel the ball towards the basket, their weaknesses in core-function, lower release height and angle could be contributing factors to reducing the likelihood of shooting success [42]. Other researchers have also acknowledged a wheelchair basketball player’s classification has the potential to, directly and indirectly, impair their shooting performance [43]. Moreover, our findings indicate teams should consider prioritising the opportunities for high-point players to attempt the field-goals, regardless of the time left on the clock or the location on the court. As our findings clearly show the superior field-goal success of high point players, it would be remiss in a disability sport in which success, for field-goal shooting at least, is now even more so clearly predicted by greater physical function, to not offer ways to re-envisage use of such classification from ability-biased, to more ‘*disability-supportive*’ to more effectively ‘*ensure parity in competition and equity in play*’ ([44], p.195). For example, limiting the number of points that can be scored per game by higher classification players, or increasing the return on a field-goal attempt by lower classification players e.g. 5 points instead of 3, or 4 points instead of 2. Sport performance analysis research in wheelchair basketball, as the only discipline to directly capture applied game related performance data, should have a unique contribution to make to such debate.

Our results suggest that the pre-shot action directly leading to the field-goal attempt is optimised following a ‘Catch & Shoot’ action, and lowest following a ‘Dribble & Shoot’. The results suggest that neither a ‘Curl’ nor a ‘Pick n Roll’ in the lead up to the shot attempt, has a better or worse impact on scoring success compared to the ‘Catch & Shoot’ action. These findings align with running basketball [45,46], whereby dribbling the ball allows for the defence to organise and apply pressure. Instead, accurate ball movement has been shown to unsettle defences leading to a quick catch and shoot action under minimal pressure, which has been found to attribute to higher shooting efficiencies. Notably, to increase the odds of field-goal success, teams should attempt to reduce the time and actions completed by a player leading up to the release of the ball, focusing on moving the ball between players to create space and optimise the ability to catch and shoot, curl or pick n roll.

Previous research regarding the relationship between player classification and core function [42] could also explain why shooting from an ‘Away From Basket’ position was found to significantly reduce shooting success compared to the baseline ‘Stationary’ category. Erčulj and Štrumbelj [47] identified the importance of avoiding this movement in running basketball to increase the likelihood of achieving shooting success. While moving away from the basket, players have been found to misjudge the depth and therefore the necessary power to propel the ball towards the basket [48]. Subsequently, as the player moves away, their base of support becomes less stable and their ability to rely on the upper extremities to provide stability reduces, along with their shooting accuracy [49]. Shooting from a moving ‘Towards Basket’ ‘Rotating Left’ or ‘Rotating Right’ did not significantly affect wheelchair basketball shooting success compared to being ‘Stationary’, indeed ‘Towards Basket’ tended towards a greater success rate possibly due to the additional propulsion generated through a moving chair especially when directly aligned with the basket. From these movements, it appears players were more able to judge the required depth and engage the shoulder joints to achieve the required vertical components of release velocity and backspin [50,51]. Therefore, making sure a player is attempting to take a shot from a stable base of support is necessary to increase the odds of field-goal success.

### Defensive implications

The findings from previous research align with the result of the present study concerning the relationship between defensive actions and field-goal attempt efficiency [48,52]. A trend was observed that as defensive pressure increased the odds of shooting success decreased (although not all significantly). In particular, our finding aligns with Tsamourtzis et al.’s [53] work, whereby the lowest field-goal shooting efficiency in running basketball was found when an offensive team is forced to attempt a shot from an increased distance away from the basket whilst in the vicinity of defensive players. Thus, defensively, teams should also seek to force the offensive team to take shots from as far from the basket as possible and failing this from either ‘elbow’ position or from the left side of the court. The data in our study also identified that having defenders covering three sides (270-degree radius) of the shooter’s sphere significantly reduced the odds of shot success, thus identifying itself as the optimum defensive strategy. This finding is added to by our results which also show that having just ‘One’ or ‘Two’ players actively involved in generating this defensive pressure provides the optimum reduction in potential shooting success. Indeed, where ‘Three’ players are involved in actively defending a field-goal attempt the chance of disrupting the attempt was shown to be less effective. This we believe identifies that when too many defenders are present (three) that their combined effect as defenders is reduced through each defender hindering the defensive effectiveness of the other. It is critical therefore that only two defenders are used to create the greatest defensive pressure as previous research within wheelchair basketball [54], basketball [55,56] and invasion games [57,58] clearly supports this reduction in skill execution as a result of increased defensive pressure.

Researchers have also found there to be a balance in optimum defensive strategies [59,60] particularly ensuring that overloading Defensive Pressure does not leave other areas of the court vulnerable to being exploited by the team in possession, i.e. those closest to the basket. Based on our findings, when defending, teams should look to restrict the speed of the ball and chair movement for the offensive team, forcing teams to take a field-goal attempt from further from the basket, later in the shot clock and from a non-stable base of support. Csataljay et al. [61] support this notion, whereby establishing optimal defensive pressure early in a possession, by affecting player and ball movement, significantly affected field-goal efficiency. Therefore, based on our findings and from a defensive perspective, teams should encourage players to defend individually or in pairs and maximise the defensive pressure that can be exerted on a shooting player by maximising their own defensive coverage and affecting the shooting player’s base of support rather seeking additional support from a third defender. This would also permit the unnecessary ‘third’ defender to defend elsewhere to reduce the availability of alternate unpressured field-goal attempts.

Further to this, the field-goal efficiency significantly reduced if a ‘Low-Pointer’ attempted the identical attempt that a ‘High-Pointer’ had made. Low-point players typically sit at a lower seating height and often in a bucketed seat to aid their base of support [43]. The assistance via the chair in core stability affects the seating height of the player, meaning a low point player has a lower release height of the ball. Subsequently, these factors in regards to a player’s functional capacity, in all three planes of movement, have been shown to reduce field-goal effectiveness as well as requiring less defensive pressure to disrupt a field-goal attempt [62]. Therefore, consideration needs to be taken by the defensive team regarding who to defend to achieve maximum disruption on a field-goal attempt. The defensive team should look to force a low-point player to handle the ball and propel the ball towards the basket on a greater number of occasions. This is even more apparent within lower-ranked teams whereby our data suggests the differences in field-goal efficiency between high and low point players is more apparent. Whilst with mid-point or high-point players the defenders should attempt to force the chair to move away from the basket, not only affecting their base of support but also their ability to correctly judge the required forces to propel the ball towards the basket [48], in an attempt to reduce field-goal attempt efficiency.

### Limitations

Of course, our study is not without its limitations. The data, although from the highest competitive level, are drawn from one competition and the process of statistical modelling is by its very nature reductionist in removing details of individual teams broader strategies when playing against the opposition of varying quality and possessing different strengths. We are also not able from our data to consider the impact of the classification total of the team line-up on shooting success and the exact classification as we only considered the classification category of the player taking the shot in the model. We were therefore not able to include in the model, offence or defence team total classification at the time a field-goal was attempted. This may be an important consideration to include in future as to how this total is comprised might influence field-goal shooting success due to its potential to enable and promote the implementation of different set plays and offensive, and indeed defensive strategies based on combined classifications of the 10 players on the court at any one time Whilst we were unable to explore three-point field goal attempts separately as distinctly different attempts to score field goals due to their relatively low frequency within the data set (339 attempts), we believe their inclusion in the model was warranted due to their occurrence as a field goal attempt and as such a sub-component of all field goal attempts that are as a result more reflective of overall game performance and subsequent match outcome.

## Conclusion

The developed unique model enables coaches, players and/or support staff to understand like never before, the influence of different variables and situations on the outcome of a field-goal shot attempt. In particular, teams should devise offensive game strategies that maximise shooting locations from the near, right 45 or base locations. There should be separate strategies for transitional offences and half-court offences based on the ability to create space for the shooting player to release the ball under minimal pressure. Within their offences, teams should focus on ball movement to reduce the need to dribble reducing the ability for the defence to establish itself and reducing the actions completed by a player leading up to the release of the ball. This ball movement aims to allow the shooting player to position their chair and establish a stable base of support, regardless of classification, before receipt of the ball and prior to releasing the ball, thus, generating the required force to propel the ball towards the basket and increasing the odds of shot success. Furthermore, when devising these offensive strategies, teams and their coaches should establish multiple effective two-man partnerships within a line-up to ensure more than one high-point shooting threat is on the court, ensuring defenders are engaged. From a defensive perspective, teams should explore ways and devise strategies whereby a single defender can cover more than 180 degrees of a player’s cylinder and exert pressure. By doing so the team can achieve maximum defensive coverage and maintain defensive balance without leaving an offensive player with an unpressured field-goal attempt. Thus, the model should be integrated within a team’s training and competition preparations by the coaches, players and/or support staff, to inform the decision making process in training and in real-time within games to potentially improve shooting effectiveness and subsequent team success. That said, the results of our study clearly identify the greater success of the least disabled players and in so doing raise further concerns regarding the effectiveness of the current classification system that needs to change in order to be more ‘*disability-supportive*’ ([44], p.195).

## Supporting information

S1 File Operational definitions for the agreed list of categories and action variables. (DOCX)
