# Supplementary material for: Predicting field-goal success according to offensive, defensive and contextual variables in elite men’s wheelchair basketball
Source: PLoS One. 2021 Jan 7;16(1):e0244257. doi: 10.1371/journal.pone.0244257 (PMC7790280; doi:10.1371/journal.pone.0244257)
Supplement: S1 File — (DOCX) [file pone.0244257.s001.docx]

# Supporting Information 1: Operational definitions for the agreed list of CPVs and action variables for the offensive, defensive and contextual characteristics.

Sup Table 1. 1: Operational definitions for the agreed list of CPVs and action variables for the offensive characteristics.

| CPV | Action Variable | Operational Definition |
| --- | --- | --- |
| Field Goal Attempt Outcome | Successful | A shot that falls through the ring and is awarded the relevant points by the referee, indicated by the number of fingers held up by his hand. |
|  | Unsuccessful | A shot that does not fall through the ring and is rebounded by a player or player is stopped due to a foul/violation or the ball goes out of bounds. |
| Classification Category | High-Pointer | The shooting player has a classification of 3.5 or above. |
|  | Mid-Pointer | The shooting player has a classification of 2.0, 2.5 or 3.0. |
|  | Low-Pointer | The shooting player has a classification of 1.0 or 1.5. |
| Number of Hands on the Ball | One Hand | When a player used his left or right hand to take a shot, with the other hand on the chair/wheel. |
|  | Two Hands | When a player used both his hands to propel the ball towards the basket. |
| Pre-Shot | Catch and Shoot | The first action following catching the ball is to make a shot attempt. |
|  | Curl | Primary threat dives into the defence and seals off, the secondary threat comes high or low with speed as the primary threat pushes the secondary threat. |
|  | Dribble and Shoot | Following possession of the ball, the player dribbles the ball and makes a shot attempt. |
|  | Pick and Roll | The creation of the pick leads to a player making an unmarked cut to the basket and a shot. |
| Shot Clock Remaining | 6 – 0.1 Seconds | The time remaining on the shot clock when the offensive player propels the ball towards the basket. The time is recorded when the ball is released from the shooting player’s hands and not when the ball hits the ring, backboard or when the basket is scored. 17 - 13 Seconds is also triggered when a player’s free-throw attempt (successful or unsuccessful) would result in the shot clock counting down from 14 seconds. |
|  | 12 - 7 Seconds |  |
|  | 17 - 13 Seconds |  |
|  | 24 - 18 Seconds |  |
| Shot Hand | Left-Handed | When a player used his left hand as the main hand to project the ball, with his right hand being used as a guide. |
|  | Right-Handed | When a player used his right hand as the main hand to project the ball, with his left hand being used as a guide. |
| Shot Location | 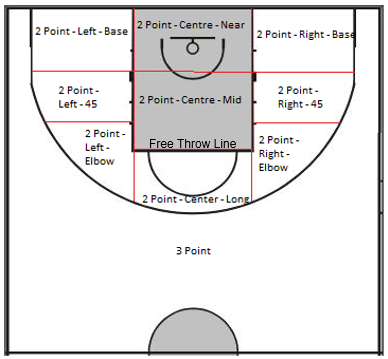  The location on the court where the shot attempt is taken from, this is measured from the position of the front castors. | |
| Shot Movement | Away from Basket | When the shooting player is extending the distance between him and the basket at the point of release. |
|  | Rotating Left | One second before and up to the point of release, the chair is rotating left increasing the angle between the basket and his release position. |
|  | Rotating Right | One second before and up to the point of release, the chair is rotating right increasing the angle between the basket and his release position. |
|  | Stationary | The player attempting to make the shot is in a stationary position and the chair is not moving at the point of release. |
|  | Towards Basket | When the shooting player is closing the gap down between him and the basket at the point of release. |
| Shot Point | Two | The ball is propelled towards the basket from inside the three-point zone and the referee will raise one hand in the air and holds up two fingers. |
|  | Three | The ball is propelled towards the basket from outside the three-point zone and the referee will raise one hand in the air and hold up three fingers. |
| Shot Positioning | 10-90 Left | At the point when the ball is released the player’s shoulders are facing an angle in the region of 10-90 degrees to the left with 0-10 degrees being Square to Basket. |
|  | 10-90 Right | At the point when the ball is released the player’s shoulders are facing an angle in the region of 10-90 degrees to the right with 0-10 degrees being Square to Basket. |
|  | Square to Basket | At the point when the ball is released the player’s shoulders are facing parallel (10-0-10 degrees) to the basket in which he is shooting. |
|  | Reverse | At the point when the ball is released the player’s shoulders are facing an angle greater than 90 degrees to the basket in which he is shooting. |
| Shot Type | Set-Shot | A shot is taken with the ball extending from the chest or shoulder towards the basket from a range of locations. |
|  | Post-Up | A shot is usually taken in or near the edge of the key, where the player holds the ball directly above their head and directs towards the basket. |
|  | Lay-Up | A player is in motion towards the basket at a 45-degree angle taking a shot, with a release point of the shoulder/head, whilst close to the basket and using the backboards. |

Sup Table 1. 2: Operational definitions for the agreed list of CPVs and action variables for the defensive characteristics.

| Category | Action Variable | Operational Definition |
| --- | --- | --- |
| Defender Behind | Yes or No | If the defending player’s chair is positioned towards the shooting player’s rear castors or backrest and is within one metre of the shooting player (see figures below) he is Defending Behind and the action is awarded a Yes. |
|  | 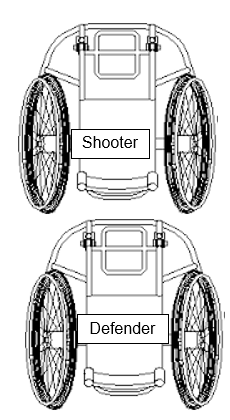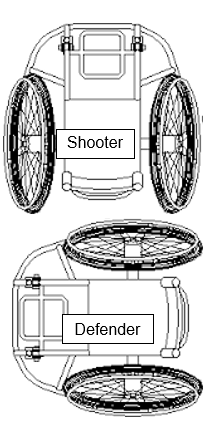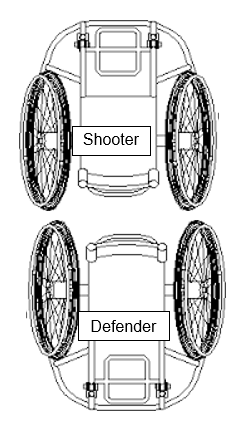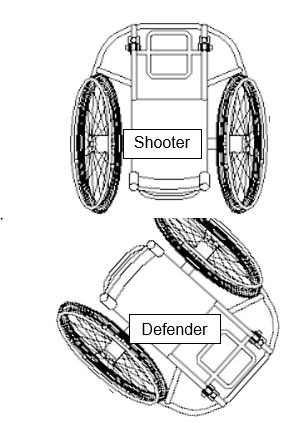 | |
| Defender In Front | Yes or No | If the defending player is positioned facing the shooting players small castors at the front of the chair and is within one metre of the shooting player (see figures below) he is defending in front and the action is awarded a Yes. |
|  | 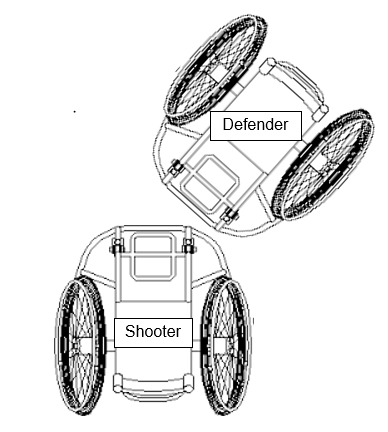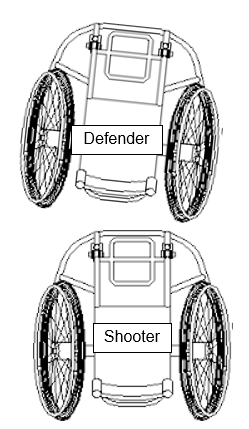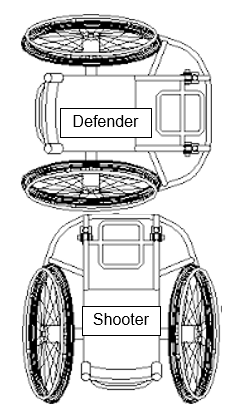 | |
| Defender Marking Shooting Hand | Yes or No | If the defending player is a chairs distances away from the player and can apply pressure to the main shooting hand through raising his hand to disrupt the vision or flight of the ball, he is Defender Marking the Shooting Hand and the action is awarded a Yes. |
| Defender Marking Non-Shooting Hand | Yes or No | The defending player is within a chairs distances away from the player and can apply pressure to the non-shooting hand through raising his hand to disrupt the vision or flight of the ball, the defender is marking the Non-Shooting Hand and the action is awarded a Yes. |
| Defender Marking Space | Yes or No | The defending player is within a chairs distances away from the player and can apply pressure by being near and is not raising his hand/s, the defender is marking the Space and the action is awarded a Yes. |
| Defender On Side | Yes or No | If the defending player’s chair is positioned towards the shooting player’s large wheel and is within one metre of the shooting player (see figures below) he is Defending On Side and the defensive action is awarded a Yes. |
|  | 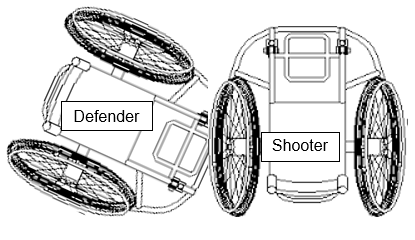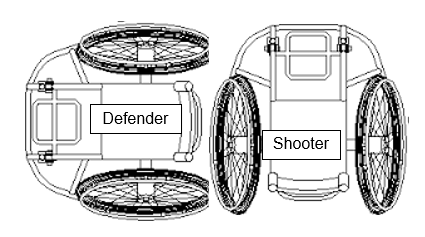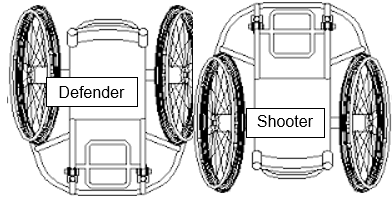 | |
| Defensive Pressure | 0-degrees | There are no defensive players within a one-metre radius of the shooting player’s sphere. |
|  | 90-degrees | The defensive player/s are using their hand/s to enter a 90-degree radius of the shooting player’s sphere. |
|  | 180-degrees | The defensive player/s are using their hand/s to enter a 180-degree radius of the shooting player’s sphere. |
|  | 270-degrees | The defensive player/s are using their hand/s to enter a 270-degree radius of the shooting player’s sphere. |
|  | 360-degrees | The defensive player/s are using their hand/s to enter a 360-degree radius of the shooting player’s sphere. |
| Number of Defenders | Zero | The number of players within one metre of the shooting player who is either making physical chair contact or visually engaged in the shooter. |
|  | One |  |
|  | Two |  |
|  | Three |  |
|  | Four |  |
|  | Five |  |

Sup Table 1. 3: Operational definitions for the agreed list of CPVs and action variables for the contextual characteristics.

| Category | Action Variable | Operational Definition |
| --- | --- | --- |
| Game Status | Winning | At the start of a possession, the team with the ball are currently leading on the scoreboard. |
|  | Drawing | At the start of a possession, the team with the ball are currently drawing on the scoreboard. |
|  | Losing | At the start of a possession, the team with the ball are currently losing on the scoreboard. |
| Quarter | Q1 | A possession which occurs during the stated quarter of the game. The time in the game is indicated on the scoreboard. Each quarter lasts 10 minutes, with the clock stopping when the ball is dead (out of bounds, foul or the referee stops play). |
|  | Q2 |  |
|  | Q3 |  |
|  | Q4 & OT |  |
